# Retraction Note: CSN6 promotes melanoma proliferation and metastasis by controlling the UBR5-mediated ubiquitination and degradation of CDK9

**DOI:** 10.1038/s41419-025-07891-8

**Published:** 2025-07-24

**Authors:** Yanli Zhang, Jianbing Hou, Shaomin Shi, Juan Du, Yudong Liu, Pan Huang, Qian Li, Lichao Liu, Huanrong Hu, Yacong Ji, Leiyang Guo, Yaqiong Shi, Yaling Liu, Hongjuan Cui

**Affiliations:** 1 Department of Dermatology, The Third Hospital of Hebei Medical University, 050051 Shijiazhuang, Hebei China; 2 State Key Laboratory of Silkworm Genome Biology, Southwest University, 400715 Chongqing, China; 3 Cancer center, Medical Research Institute, Southwest University, 400716 Chongqing, China; 4 Chongqing Engineering and Technology Research Centre for Silk Biomaterials and Regenerative Medicine, 400716 Chongqing, China; 5 Engineering Research Center for Cancer Biomedical and Translational Medicine, Southwest University, 400716 Chongqing, China

Retraction Note to: *Cell Death & Disease* 10.1038/s41419-021-03398-0, published online 22 January 2021

The Editors-in-Chief have retracted this article. After publication, the authors found some errors in the images presented in Figs. 2 and 5. Further checks by the publisher identified additional similarities in a number of blots and cell images in Figs. 2–5.

The Editor-in-Chief therefore no longer has confidence in the presented data.

The corresponding author has stated on behalf of all authors that they agreed with this retraction, but not the wording of this retraction notice.

